# Phytochemical Analysis of *Veratrum* Alkaloids in Medicinal *Veratrum* Globules Using High‐Performance Liquid Chromatography Coupled With Tandem Mass Spectrometry

**DOI:** 10.1002/bmc.70380

**Published:** 2026-02-06

**Authors:** Julia Siegle, Jörg Pietsch

**Affiliations:** ^1^ Institute of Legal Medicine, Medical Faculty Carl Gustav Carus Technical University Dresden Dresden Germany

**Keywords:** globules, HPLC–MS/MS, jervine, veratramine, *Veratrum*

## Abstract

*Veratrum* L. species have been used in traditional medicine for centuries due to their bioactive properties, yet they also contain toxic steroid alkaloids that pose potential health risks when present in herbal preparations. Reliable analytical approaches are therefore essential to ensure product safety, protect consumer health and assess toxicological evaluations. This study presents the development, validation, and application of a highly sensitive analytical method to determine alkaloid levels in commercially available *Veratrum* globules. Five *Veratrum* alkaloids (cevadine, jervine, protoveratrine A, veratramine, and veratridine) were analyzed in 10 different *Veratrum* globules ( *Veratrum album* L.: D2, D3, D4, D6, D12, D30, D200; *Veratrum viride var. viride*: D6, D12, D30) utilizing high‐performance liquid chromatography coupled with tandem mass spectrometry (HPLC–MS/MS). Jervine contents were successfully quantified in *V. album* L. globules with decreasing concentration in potencies D2 (25 ng/g), D3 (2 ng/g) and D4 (0.2 ng/g). These findings demonstrate that the applied method enables the precise identification and quantification of toxic alkaloids in highly diluted preparations. The results contribute significant data for the toxicological assessment of herbal products and highlight the critical role of advanced analytical control in ensuring the safety and quality of herbal medicines, thereby helping to prevent potential health risks to consumers.

**Abbreviations**
ce
: Collision energy
CXP: Cell exit potential and measurement time
DAD: Diode array detector
DP: Declustering potential
EP: Entrance potential
ESI: Electrospray ionization
FDA: Food and Drug Administration
GHP: German Homoepathic Pharmacopoeia
GTFCh: Gesellschaft für Toxikologische und Forensische Chemie
HAB: Homöopathisches Arzneibuch
HMA: Heads of Medicines Agencies
HMPWG: Homeopathic Medicinal Products Working Group
HPLC: High‐performance liquid chromatography
HPTLC: High‐performance thin layer chromatography
HPUS: Homeopathic Pharmacopoeia of the United States
ICH: International Council for Harmonisation of Technical Requirements for Pharmaceuticals for Human Use
LOD: Limit of detection
LOQ: Limit of quantification
MRM: Multiple reaction monitoring
MS: Mass spectrometry
MS/MS: Tandem mass spectrometry
R^2^
: correlation coefficient
RDA: retro‐Diels–Alder
RSD: Relative standard deviation
SD: Standard deviation
UV: Ultraviolet
WHO: World Health Organization

## 1 Introduction

The medicinal significance of the *Veratrum* L. genus, belonging to the *Melanthiaceae* family (*Liliales* order), has long been known from historical records of traditional Chinese medicine, ancient Egypt, the Celts, and Romans (Wink et al. 2008; Zhao et al. 2024). *Veratrum* L. plants contain various toxic steroid alkaloids, such as protoveratrine A, jervine, and veratramine, which are formed from the precursor molecule cholesterol (Chandler and McDougal 2014). These alkaloids bind to sodium voltage‐gated channels, building action potentials in cardiac, nerve, and skeletal muscle cells (Chen et al. 2019; Schep et al. 2006; Wendt et al. 2022). Consequently, the cells have a higher permeability to sodium and calcium ions, causing a delayed neuronal repolarization and multiple discharges from a single stimulus (Schep et al. 2006). Due to their antihypertensive, anti‐inflammatory, and antioxidant properties, *Veratrum* alkaloids are promising bioactive compounds demonstrating therapeutic potential, particularly in oncology and cardiology (Dumlu et al. 2019; Lei and Huo 2020; Li et al. 2006; Seale and McDougal 2022; Zhou et al. 2023). Traditionally, *Veratrum* preparations have been used in various forms of herbal medicine and homeopathy to treat a variety of symptoms, such as vomiting, hypertension, epilepsy, or blood‐stroke (Chen et al. 2019; Van Wassenhoven 2004).

The term “homeopathy” is derived from the Greek words “homoios” meaning same (or similar) and “pathos” standing for suffering (Fisher 2012). It represents an application of complementary and integrative medicine founded by Samuel Hahnemann in 1796 based on the principle of “similia similibus curentur”, meaning “like cures like” (Fisher 2012; World Health Organization 2009). Worldwide, over 200 million people regularly utilize homeopathic products, which are integrated into the national healthcare systems of several countries, such as Brazil, India, and Switzerland (Leemhuis and Seifert 2024). Homeopathic medicines are prepared mainly from plant or animal substances based on the local Homeopathic Pharmacopoeia (Pharmacopoeia Europaea, Ph. Eur.; “Homöopathisches Arzneibuch”, HAB used in this study), which includes guidelines for the preparation, potentization and labeling of mother tinctures (Bundesinstitut für Arzneimittel und Medizinprodukte 2023). Therefore, highly diluted substances in the form of globules and other preparations, including tablets, tinctures, dilutions or ointments, are employed to treat a variety of ailments (Şenel 2019). Globules are sugar‐based spherical carriers composed of pharmaceutical‐grade sucrose and are produced through a controlled granulation process to ensure uniformity in size and mechanical stability (Bundesinstitut für Arzneimittel und Medizinprodukte 2023). These sucrose beads are either impregnated with or coated by highly diluted plant‐based or other active preparations according to specific manufacturing guidelines (Bundesinstitut für Arzneimittel und Medizinprodukte 2023). For instance, the HAB regulation no. 10 describes the impregnation of neutral globules with a liquid preparation in a typical ratio of 1:100 (drug to carrier by mass), while regulations no. 39a‐c outline methods for coating globules with combinations of triturated or liquid active substances mixed with sugar syrup (Bundesinstitut für Arzneimittel und Medizinprodukte 2023). The finished product is then labeled with the dilution stage corresponding to the applied preparation. These procedures are standardized and regulated to ensure consistent product quality (Bundesinstitut für Arzneimittel und Medizinprodukte 2023). For each plant, an individual HAB monograph describes the raw material, preparation methods of the herbal mother tincture, analytical tests, assays, and storage conditions (Bundesinstitut für Arzneimittel und Medizinprodukte 2023; Colalto 2020). However, these guidelines do not serve to ensure the safety of homeopathic products but are intended to guarantee a certain quality of homeopathic preparations (Colalto 2020; Vikram et al. 2024). The tests and assays of the German Homoeopathic Pharmacopoeia (GHP equals HAB) rely on analytical methods (e.g., thin layer chromatography) for individual substances and do not account for the multitude of bioactive compounds present in the products (Bundesinstitut für Arzneimittel und Medizinprodukte 2023). In Europe, the Homeopathic Medicinal Products Working Group (HMPWG), established by the Heads of Medicines Agencies (HMA), deals with the regulation and standardization of homeopathic medicines for human and veterinary use. The publication of harmonized guideline documents by the HMPWG aims to facilitate the exchange of regulatory and scientific expertise for the evaluation of the production, quality, safety, and use of homeopathic medicines (Habs and Koller 2021; Homeopathic Medicinal Products Working Group 2016, 2020). Moreover, in efforts to standardize the quality, safety and efficacy of homeopathic products, the World Health Organization (WHO) outlines regulations and recommendations for the production and quality of such medicines (World Health Organization 2009). Previous incidents and the efforts of safety control indicate a growing regulatory focus on homeopathy and the need for improvement and expansion of sensitive analytical methods. Although various HPLC methods have been employed to analyze especially homeopathic tinctures (Chaudhari and Mashru 2017; Girin et al. 1993; Kaya and Melzig 2008; Patel and Mashru 2017; Shukla et al. 2021; Soudagar et al. 2023), products such as globules, which are more frequently consumed by users, have remained largely unexplored.

Various errors can occur in the production of homeopathic medicines, posing a safety risk to the health of consumers (Habs and Koller 2021; World Health Organization 2009). Firstly, it is important to note the significant variations in active ingredient levels in plants, influenced by numerous factors including location, species, season, weather, and nutrients (World Health Organization 2009). Previous studies have already identified adulterations or contaminations of homeopathic products using HPLC coupled to MS, with additional or different preservatives being identified compared to those listed on the label (Panusa and Gagliardi 2008). Apart from impurities, microbial contaminations have also been found in homeopathic products, indicating improper manufacturing practices (Food and Drug Administration U.S. 2018; World Health Organization 2009). Another issue in the production of homeopathic medicines can arise from the dilution of mother tinctures, which can have serious health consequences for consumers. In September 2016, the Food and Drug Administration (FDA) issued a warning against the use of homeopathic teething aids in the form of tablets and gels containing *Atropa belladonna* L. alkaloids (atropine and scopolamine), following reports linking the products to severe adverse events such as seizures and deaths in infants and children (Food and Drug Administration U.S. 2017b). Subsequent FDA laboratory LC‐MS analyses confirmed in 2017 that certain homeopathic products contained elevated and inconsistent levels of caffeine (from unknown *Coffea* L. cruda) as well as atropine and scopolamine (Food and Drug Administration U.S. 2017a; Habs and Koller 2021). To enhance the safety and quality of homeopathic medicines, the FDA handed over guidelines to the Homeopathic Pharmacopoeia of the United States (HPUS) in December 2022 (Food and Drug Administration U.S. 2022).

A toxicological assessment of the poisoning potential arising from homeopathic products is of great importance. Calculation approaches for the risk assessment have been developed from results of available controlled clinical studies of homeopathic products or data of the corresponding Pharmacopoeia (Buchholzer et al. 2014; Habs and Koller 2021). However, various challenges arise due to the multitude and diversity of raw materials used, the different formulations, and the limited available toxicological data (Buchholzer et al. 2014). Buchholzer et al. have compiled extensive toxicological assessment data of homeopathic and anthroposophical medicines (Buchholzer et al. 2019; Buchholzer et al. 2022), with the approaches to calculating “safe” daily doses being contentious and subject to discussion (Nersesyan 2022). These discrepancies in toxicological assessment can be scrutinized through precise analytical investigations and quantifications of the bioactive compounds in homeopathic medicines. Particularly vulnerable people like young children, the elderly, and pregnant or breastfeeding women should be considered in the toxicological assessments (World Health Organization 2009).

In previous investigations of *Veratrum* alkaloid contents in three distinct *Veratrum* species, a precise analytical method for the determination of the alkaloids, including cevadine, jervine, protoveratrine A, veratramine, and veratridine, was established (Siegle and Pietsch 2024). The developed HPLC‐MS/MS method is adjustable and applicable for *Veratrum*‐containing homeopathic products, as former research also utilized liquid chromatography and mass spectrometry (Food and Drug Administration U.S. 2017a; Panusa et al. 2007; Siegle and Pietsch 2024). Previous research in homeopathy has primarily focused on applications and efficacy (Kumar et al. 2021; Mohan et al. 2022), while analytical investigations of plant‐based active ingredients in homeopathic products have been limited.

This study presents, for the first time, a comprehensive analytical investigation of plant‐derived active substances within the matrix of globules, using a highly sensitive and validated HPLC‐MS/MS method. The study addresses an unexplored area by detecting and quantifying *Veratrum* alkaloids at extremely low‐concentration levels relevant for medicinal products and homeopathic preparations. The aim is to develop, validate, and apply an accurate analytical approach that provides precise data on the individual active compound contents in globules. A toxicological assessment of the studied globules focused on pediatric safety was also included, as this is particularly relevant for poison control centers and the risk evaluation for children.

## 2 Materials and Methods

### 2.1 Chemicals and Reagents

For the mobile phase of the HPLC, formic acid from Merck KGaA (Darmstadt, Germany), ammonium formate from Fluka Analytical (Steinheim, Germany), HPLC grade acetonitrile from VWR International (Rosny‐sous‐Bois‐cedex, France), and HPLC grade water from Fisher Scientific (Loughborough, UK) were purchased. The 2‐propanol for the rinsing solution was obtained from Honeywell (Seelze, Germany). Atropine‐d_3_ (100 μg/mL) was purchased from Sigma Aldrich (Steinheim, Germany) and applied as internal standard with a concentration of 10 ng/μL (Siegle and Pietsch 2024).

### 2.2 Globules Material

*Bryonia cretica* L. D6 globules (10 g) from DHU‐Arzneimittel GmbH & Co. KG (Karlsruhe, Germany) were used as analyte‐free matrix for the calibration. The *Veratrum* globules samples (each 10 g) from DHU‐Arzneimittel GmbH & Co. KG (Karlsruhe, Germany) were selected according to their commercial availability. Globules of two different *Veratrum* species in varying potencies ( *Veratrum album* L.: D2, D3, D4, D6, D12, D30, D200; *Veratrum viride var. viride*: D6, D12, D30) were obtained from the pharmacy. According to the patient information leaflet, five globules (one dose) should be taken three times daily. Therefore, in the following procedures, five globules were the standard amount for the measurements.

### 2.3 Sample Preparation

Five globules (approximately 45 mg) were solved in 95 μL of mobile phase (A:B, 90:10) and 5 μL of atropine‐d_3_ (10 ng/μL). After 5 min of shaking and 1 min of centrifugation, the dissolved globules are measured applying the MRM method for *Veratrum* alkaloids. Globules samples were prepared in the same manner for all measurements of the method validation with *Veratrum*‐free *Bryonia cretica* L. D6 globules (calibration, linear range, analytical limits, recovery, stability, accuracy, precision) and quantification with the *Veratrum* globules. For each HPLC measurement, 20 μL of the investigated sample is injected.

Five globules weigh approximately 45 mg; all further experiments were calculated for clarity from the unit “pg/45 mg globules” into the SI unit “ng/g globules”.

### 2.4 Standard Solutions

Reference standards of the investigated *Veratrum* alkaloids, including cevadine, jervine, protoveratrine A, veratramine, and veratridine, were purchased from PhytoLab GmbH & o. KG (Vestenbergsgreuth, Germany) (Siegle and Pietsch 2024). Stock solutions with the concentration of 1.0 mg/mL were prepared for each alkaloid standard in ethanol, which was obtained from Fisher Scientific (Loughborough, UK). Standard solutions with concentrations between 100 and 0.001 ng/μL were received by mixing and diluting the alkaloid stock solutions (Siegle and Pietsch 2024)

### 2.5 HPLC‐MS/MS Conditions

Each analysis was accomplished by the 1260 Infinity Quaternary Pump HPLC System from Agilent Technologies (Santa Clara, California, USA) coupled with the 3200 QTRAP tandem mass spectrometer from AB Sciex (Darmstadt, Germany). On the Luna Pentafluorophenyl (2) column (100 Å, 150 mm × 2 mm, 5 μm) from Phenomenex (Aschaffenburg, Germany), the chromatographic separation was carried out within 22 min. While C18 columns rely predominantly on hydrophobic interactions, the pentafluorophenyl phase additionally provides π–π interactions, dipole–dipole interactions, and hydrogen bonding with polar functional groups of the analytes. This results in higher shape selectivity, improved separation of positional and stereoisomers, and enhanced interaction with the π‐systems of the steroidal alkaloid skeletons due to the electron‐deficient aromatic surface of the pentafluorophenyl material. The mobile phase eluents consisted of HPLC grade water (eluent A) or acetonitrile (eluent B) with 2 mM of ammonium formate and 0.2% formic acid. The gradient elution was conducted using a flow rate of 0.5 mL/min starting with an initial ratio of 90:10 (A:B), which changed in 10 min to 10:90 (A:B). Then, a flow rate of 1.0 mL/min was applied with 10:90 (A:B) for 5 min. Subsequently, the composition returned to 90:10 (A:B) in 1 min employing a flow rate of 0.5 mL/min until 22 min. For the rinsing, a 10% 2‐propanol solution diluted with HPLC grade water was utilized (Siegle and Pietsch 2024).

The ion source used by the mass spectrometer was an electrospray ionization (ESI) operating in positive‐ion mode. The nebulizer gas (45 psi), the heater gas (90 psi), and the curtain gas (35 psi) were set to the specific values, and the collision gas was on “medium” settings. The ion source temperature of 660°C and ion spray voltage of 5500 V were applied. By using a developed multiple reaction monitoring (MRM) method, the mass spectrometer detected the *Veratrum* alkaloid mass transitions as described in the previous study (Siegle and Pietsch 2024).

### 2.6 Method Development

Detecting several distinct analytes within a single sample preparation can be achieved by the development of a multimeasurement method. Given the variability of alkaloids across distinct species of *Veratrum* plants, a selection of five commercially available *Veratrum* alkaloids (cevadine, jervine, protoveratrine A, veratramine, veratridine) was implemented in the MRM measurement method for this study. As internal standard, atropine‐d_3_ was selected to ensure the quality and to compensate for matrix effects and analyte losses. The quantifier and qualifier mass transitions of each analyte were selected by measuring the stock solution of each analyte in full scan mode. The resulting mass spectra were analyzed, and the molecular ion peak and the two most intense fragment ions, quantifier, and qualifier were selected and confirmed by previous literature. Additionally, optimization of the mass transition was carried out by infusion of the stock solutions into the mass spectrometer. Subsequently, the four MS parameters, declustering potential (DP), entrance potential (EP), collision energy (CE), and cell exit potential (CXP), were tuned manually.

### 2.7 Method Validation

The validation of the developed HPLC‐MS/MS method was accomplished for parameters including the linearity, limit of detection (LOD), limit of quantification (LOQ), stability, recovery, accuracy, and precision in accordance with previous literature (Peters et al. 2007) and guidelines of the Society of Toxicological and Forensic Chemistry (GTFCh, “Gesellschaft für Toxikologische und Forensische Chemie”) (Peters et al. 2009; Schmitt et al. 2005) and the International Council for Harmonisation of Technical Requirements for Pharmaceuticals for Human Use (ICH) (International Council for Harmonisation of Technical Requirements for Pharmaceuticals for Human Use 2022). The evaluation of the validation data was carried out using the Excel‐based program VALISTAT 2.0 from ARVECON GmbH (Schmitt et al. 2005).

The linear range was examined by linear regression (correlation coefficient *R* ^2^ > 0.99) with the area ratios of seven concentration levels that were measured with at least two replicates (Peters et al. 2007). The concentration levels were prepared by solving five *Veratrum*‐free *Bryonia* D6 globules in standard solution (x μL) of the analytes, mobile phase (95‐x μL), and internal standard (5 μL) according to the description in the Sample preparation section.

The determination of the analytical limits was performed in accordance with the statistical approach outlined in DIN 32645 (Schmitt et al. 2005), rather than the commonly applied signal‐to‐noise ratio method. For this purpose, six low‐concentration calibration levels were prepared analogously to the samples used for linearity and analyzed using the MRM method. The resulting concentrations and corresponding area ratios of the quantifier MRM transitions were subjected to linear regression analysis (*R* ^2^ > 0.99). Based on the regression data, the LOD and LOQ were calculated using the VALISTAT 2.0 software in accordance with the GTFCh guidelines and DIN 32645 (Schmitt et al. 2005).

The stability of the analytes and internal standard was investigated by measuring a sample (11 ng/g) six times over a long time period. The range of the measured peak areas was calculated by the VALISTAT 2.0 software (Schmitt et al. 2005) with the Equation (1).

(eq 1) $$\text{Range}\;\left[\%\right]=\frac{{\text{peak area}}_{\max}-{\text{peak area}}_{\min}}{{\text{peak area}}_{\text{average}}}\cdot 100\%$$

Concerning the determination of the recovery, two concentration levels of the *Veratrum* alkaloids were examined. Therefore, six blank and six matrix (*Bryonia* D6 globules) samples with concentrations of 1.1 ng/g (equals 50 pg/45 mg globules) and 11 ng/g (equals 500 pg/45 mg globules) were prepared and measured. The recovery of the analytes and internal standard was evaluated according the following Equation (2) using VALISTAT 2.0 (Schmitt et al. 2005).

(eq 2) $$\text{Recovery}\;\left[\%\right]=\frac{{\text{response factor}}_{\text{matrix sample}}}{{\text{response factor}}_{\text{blank}\;\mathrm{sa}m\mathrm{ple}}}\cdot 100\%$$

The precision and accuracy of the *Veratrum* MRM method were investigated with intraday and interday experiments (International Council for Harmonisation of Technical Requirements for Pharmaceuticals for Human Use 2022; Peters et al. 2007, 2009; Schmitt et al. 2005). Eight intraday samples of two concentrations (low: 1.1 ng/g, medium: 11 ng/g) were prepared with *Bryonia* D6 globules and measured on the same day to determine the repeatability. In contrast, the interday samples of two concentrations (low and medium) were measured on eight consecutive days to examine the reproducibility of the developed HPLC‐MS/MS method. For the precision, the calculation of the relative standard deviation (RSD) of the intraday and interday experiments was conducted by application of the subsequent Equation (3).

(eq 3) $$\mathrm{RSD}\left[\%\right]=\frac{{\text{standard deviation}}_{\text{measured concentrations}}}{{\text{average}}_{\text{measured concentrations}}}\cdot 100\%$$

The accuracy was evaluated with the bias of the intraday and interday measurements which were determined as following with Equation (4).

(eq 4) $$\text{Bias}\;\left[\%\right]=\frac{{\text{mean concentration}}_{\text{measured}}-{\text{concentration}}_{\text{true}}}{{\text{concentration}}_{\text{true}}}\cdot 100\%$$

### 2.8 Calibration and Quantification

For the calibration of the globule samples, *Veratrum*‐free *B. cretica* L. D6 globules were used as calibration matrix. To guarantee the absence of *Veratrum* alkaloids in the *B. cretica* L. globules, a blank sample was examined.

Seven concentrations of the mixed alkaloid standards were prepared in the range of 0.02 to 22.2 ng/g globules (equals 1–1000 pg/45 mg globules) and measured by the developed MRM method. A calibration curve was plotted by linear regression (*R* ^2^ > 0.99).

A reliable quantification of the analytes was done above the LOQ, while analyte concentrations between the LOD and LOQ are affirmed positive.

Samples with analyte concentration above the linear range were diluted and measured again to obtain a concentration within the calibration. The resulting concentration was extrapolated to the real analyte concentration of the analyzed sample.

## 3 Results and Discussion

**FIGURE 1:**
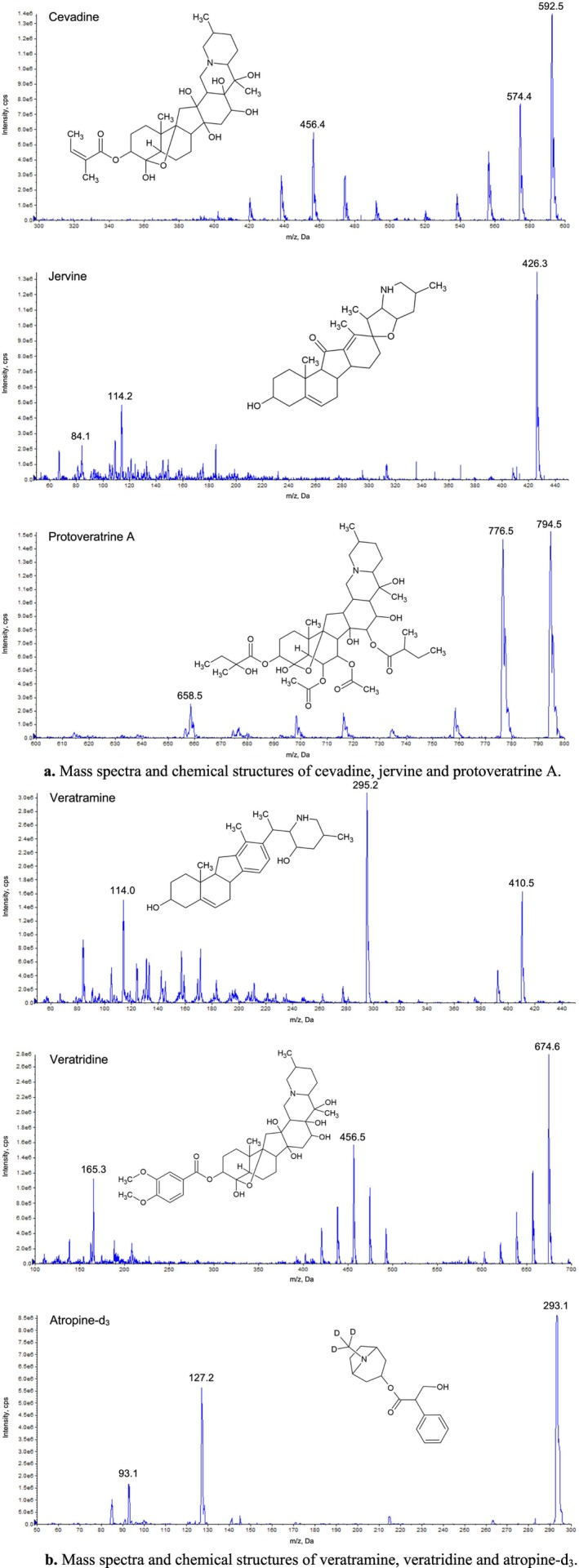
(a) Mass spectra and chemical structures of cevadine, jervine, and protoveratrine A. (b) Mass spectra and chemical structures of veratramine, veratridine, and atropine‐d_3_.

The method development was successfully completed. The mass spectra of the analytes and the internal standard recorded by full‐scans and their corresponding structures are presented in Figure 1a,b.

The investigated precursor [M + H]^+^, quantifier, and qualifier ions, along with the optimized MS parameters for each *Veratrum* alkaloid and the internal standard, are summarized in Table 1.

**TABLE 1:** Mass transitions and optimized mass spectrometer parameters of each *Veratrum* alkaloid and the internal standard.

Analyte	Precursor ion (Da)	Quantifier (Da) Qualifier (Da)	DP (V)	EP (V)	ce (V)	CXP (V)	Time (ms)
Cevadine	592.5	574.4	90	12	45	6	100
		456.4	90	12	60	4	100
Jervine	426.3	114.2	80	12	55	2	50
		84.1	70	12	60	2	50
Protoveratrine A	794.5	776.5	80	12	50	6	50
		658.5	90	12	60	6	50
Veratramine	410.5	295.2	60	12	25	4	50
		114.0	70	12	50	2	50
Veratridine	674.6	456.5	90	12	62	4	50
		165.3	90	12	80	2	100
Atropine‐d_3_	293.1	127.2	56	5	35	4	20
		93.1	56	5	43	4	20

Abbreviations: CE, collision energy; CXP, cell exit potential and measurement time; DP, declustering potential; EP, entrance potential.

**FIGURE 2:**
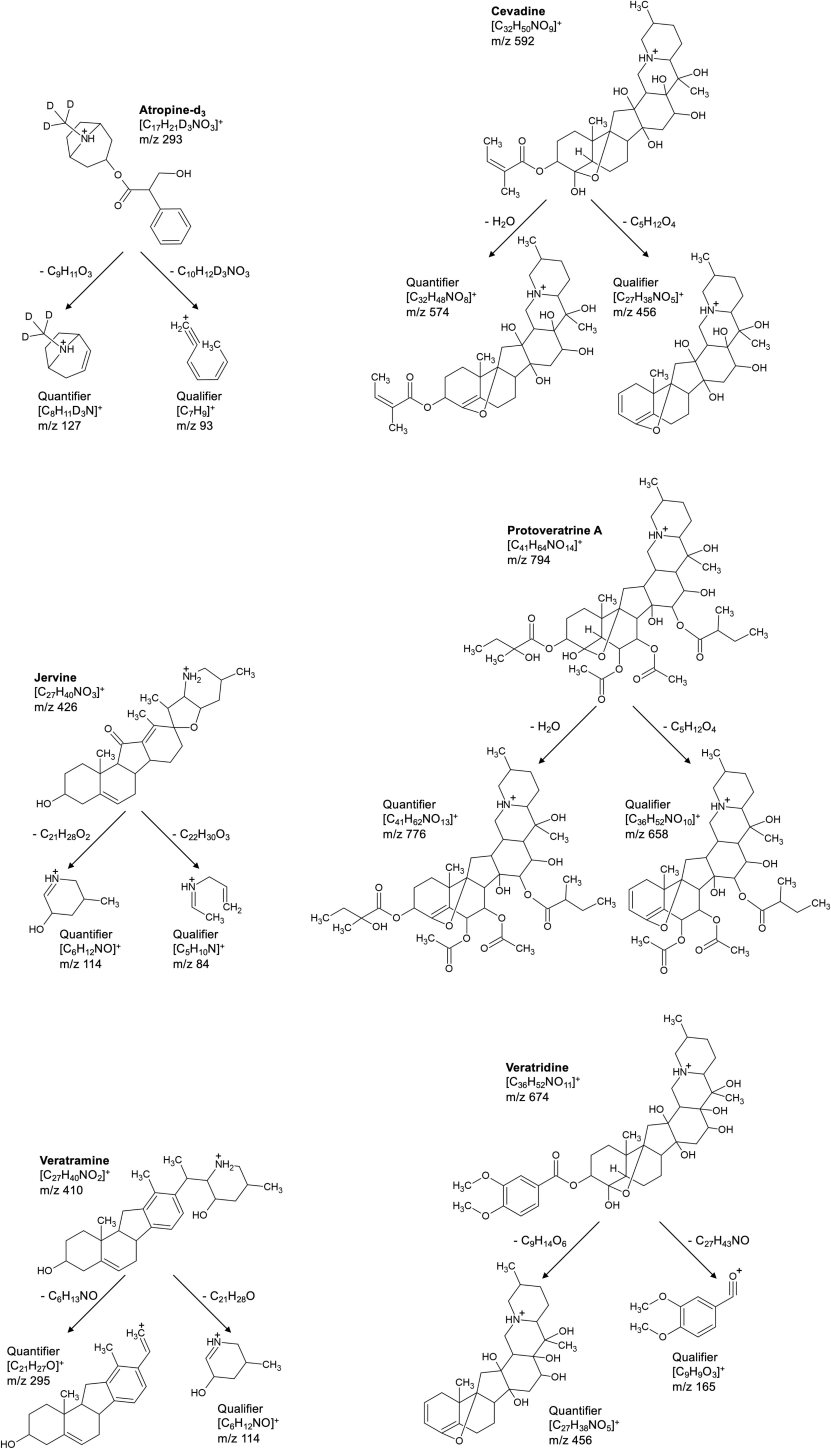
Proposed fragmentation mechanisms of atropine‐d_3_ and the *Veratrum* alkaloids.

Figure 2 illustrates the proposed fragmentation mechanisms of the *Veratrum* alkaloids and the internal standard. Due to mesomeric effects, the localization of bonds, hydrogen atoms, and the positive charge may vary between different resonance structures; therefore, only one representative fragmentation pathway is depicted for each compound.

Fragmentation of atropine‐d₃ proceeds via ester bond cleavage to form the quantifier ion (m/z 293 → 127), corresponding to the characteristic 8‐azabicyclo[3.2.1]octane structure common for tropane alkaloids (Reina et al. 2010; Zhou et al. 2017). Subsequent cleavage of the ester residue accompanied by charge‐driven rearrangement yields the qualifier ion (m/z 293 → 93).

The similar fragmentation behavior of cevadine and protoveratrine A is characterized by an initial dehydration at the A ring, resulting in the respective quantifier ions (cevadine m/z 592 → 574; protoveratrine A m/z 794 → 776), followed by cleavage of the ester bond at the A ring producing the qualifier ions (cevadine m/z 592 → 456; protoveratrine A m/z 794 → 658) (Li et al. 2007).

The quantifier ion of jervine (m/z 426 → 114) corresponds to a characteristic iminium ion formed via charge‐directed α‐cleavage adjacent to the tertiary nitrogen and subsequent rearrangement involving the E ring of the steroidal backbone (Chen et al. 2019). Further fragmentation of this iminium ion leads to the formation of the smaller qualifier ion (m/z 426 → 84). Although such fragmentation patterns are sometimes attributed to retro‐Diels–Alder (RDA) reactions, a genuine RDA reaction requires a six‐membered ring containing a double bond and yields a (substituted) diene and a (substituted) ethene (Niessen and Correa C. 2017; Tureček and Hanuš 1984). Since the observed fragmentation of jervine occurs at a five‐membered heterocyclic ring lacking a double bond, an RDA mechanism can be excluded.

Veratramine fragments predominantly via charge‐directed α‐cleavage adjacent to the nitrogen‐containing ring (Chen et al. 2019), resulting in the parallel formation of the quantifier ion (m/z 410 → 295) and the qualifier ion (m/z 410 → 114). The latter corresponds to the same characteristic iminium ion observed as the quantifier ion of jervine.

In veratridine, ester bond cleavage combined with dehydration at the A ring leads to the formation of the quantifier ion (m/z 674 → 456), which is structurally related to the qualifier ion of cevadine. The qualifier ion of veratridine (m/z 674 → 165) corresponds to an aromatic acylium‐ion fragment derived from the previously cleaved ester residue and formed via further bond cleavage and charge‐driven rearrangement.

The observed fragment ions are consistently explained by charge‐directed α‐cleavage, dehydration, and ester bond cleavage. Although RDA reactions and McLafferty rearrangements are frequently invoked for steroidal systems, neither mechanism applies here due to the absence of an unsaturated six‐membered ring at the cleavage site (RDA) and the lack of a suitable carbonyl–γ‐hydrogen geometry required for the respective concerted transition states (McLafferty).

**FIGURE 3:**
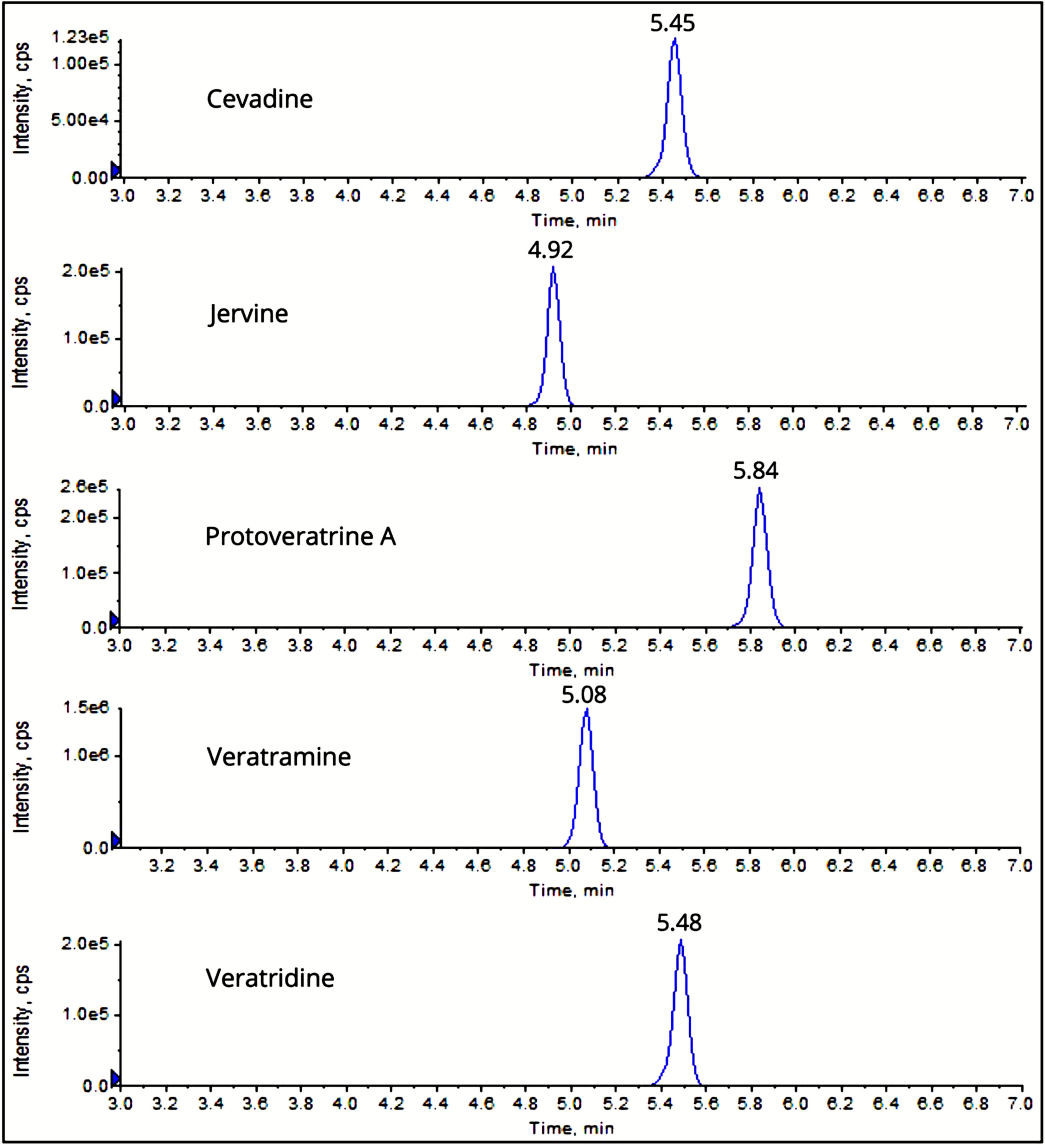
Chromatographic peaks and retention times of each quantifier mass transition measured by the developed MRM method.

Chromatographic peaks of the five *Veratrum* alkaloids analyzed using the developed and validated MRM method are shown in Figure 3.

The HPLC‐MS/MS method was validated with respect to linearity, analytical limits, stability, recovery, accuracy, and precision. Calibration was performed in the concentration range of 0.2–22.2 ng/g, yielding correlation coefficients *R* ^2^ greater than 0.995. The results of the calibration curves, linearity, and analytical limits are summarized in Table 2. Among the analyzed compounds, the linearity ranges were between 1.0 and 22.2 ng/g, with jervine showing the lowest LOD at 0.89 ng/g. Stability (11 ng/g) ranges and recovery rates (1.1 ng/g and 11 ng/g) for the *Veratrum* alkaloids and the internal standard atropine‐d_3_ are compiled in Table 3. According to the recommendations of the GTFCh (Peters et al. 2009), the variation in peak area during the stability assessment should not exceed ±25% over the observation period. This applies for all alkaloids and atropine‐d_3_, confirming the temporal stability of the analytes (11 ng/g) in the autosampler for a period of 616 min. In accordance with the GTFCh guidelines (Peters et al. 2009), each analyte should exhibit a recommended recovery over 50%. This requirement meets with all recovery rates for both examined concentrations. Notably, even at concentrations below the LOQ (1.1 ng/g), all analytes showed sufficient recoveries. Intraday and interday experiments were conducted to evaluate the accuracy and precision of the method. Results for two concentrations (1.1 ng/g and 11 ng/g globules), including the mean with standard deviation (SD), bias and RSD, are presented in Table 4. In line with GTFCh guidelines (Peters et al. 2009), acceptable accuracy and precision are defined by bias and RSD values below 15%. This requirement was fulfilled for all *Veratrum* alkaloids at both concentration levels.

**TABLE 2:** Calibration, linear range and analytical limits of the *Veratrum* alkaloids in globules matrix.

Analyte	Calibration curve	Linear range (ng/g)	*R* ^2^	LOD (ng/g)	LOQ (ng/g)
Cevadine	*y* = 1.75 exp‐2 x + 2.14 exp‐5	1.0–22.2	0.999	1.22	4.36
Jervine	*y* = 1.85 exp‐2 x + 1.36 exp‐5	1.0–22.2	0.9995	0.89	3.22
Protoveratrine A	*y* = 2.93 exp‐2 x + 6.80 exp‐6	1.0–22.2	0.9988	1.20	4.22
Veratramine	*y* = 1.92 exp‐1 x + 8.62 exp‐5	1.0–22.2	0.9994	1.07	3.82
Veratridine	*y* = 2.90 exp‐2 x + 5.59 exp‐5	1.0–22.2	0.9984	1.36	4.76

Abbreviations: LOD, limit of detection; LOQ, limit of quantification.

**TABLE 3:** Stability (11 ng/g) and recovery (1.1 ng/g and 11 ng/g) of the *Veratrum* alkaloids and atropine‐d_3_ in globules matrix.

Analyte	Stability	Recovery (%)	
	Range (%)	1.1 ng/g	11 ng/g
Cevadine	18	71	92
Jervine	16	58	90
Protoveratrine A	22	67	89
Veratramine	15	62	91
Veratridine	11	74	91
Atropine‐d_3_	23	82	99

**TABLE 4:** Accuracy and precision results of interday and intraday experiments for analyte concentrations of 1.1 ng/g and 11 ng/g.

	Interday			Intraday		
	Mean ± SD	Accuracy	Precision	Mean ± SD	Accuracy	Precision
	(ng/g)	Bias (%)	RSD (%)	(ng/g)	Bias (%)	RSD (%)
1.1 ng/g						
Cevadine	1.1 ± 0.1	−2.0	6.8	1.1 ± 0.1	0.6	8.8
Jervine	1.1 ± 0.2	−1.0	13.6	1.2 ± 0.1	3.3	9.7
Protoveratrine A	1.0 ± 0.1	−7.5	11.9	1.1 ± 0.0	1.3	3.0
Veratramine	1.2 ± 0.1	6.0	5.4	1.1 ± 0.1	2.2	5.1
Veratridine	1.0 ± 0.1	−9.1	11.0	1.2 ± 0.1	7.3	5.3
11 ng/g						
Cevadine	11.2 ± 0.8	1.2	6.7	11.1 ± 0.4	−0.2	3.9
Jervine	11.2 ± 0.6	1.1	5.7	11.3 ± 0.5	1.7	4.0
Protoveratrine A	11.4 ± 0.7	2.2	5.7	11.2 ± 0.3	1.2	2.5
Veratramine	11.4 ± 0.6	2.3	5.6	11.1 ± 0.3	0.3	2.3
Veratridine	11.2 ± 0.5	0.4	4.8	11.0 ± 0.8	−1.0	7.0

Abbreviations: RSD, relative standard deviation; SD, standard deviation.

**FIGURE 4:**
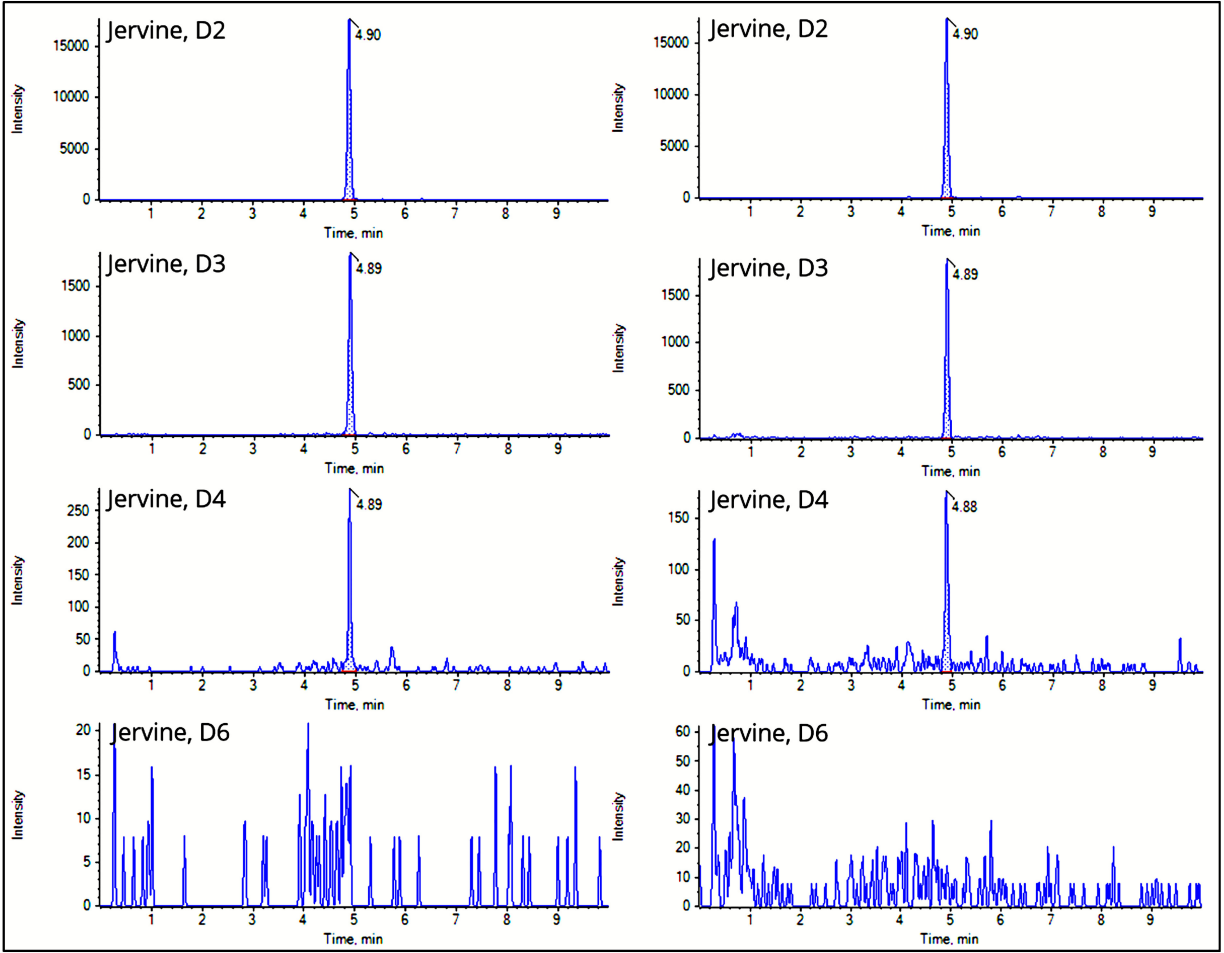
Chromatograms of jervine quantifier (m/z 426 → 114, left) and qualifier (m/z 426 → 84, right) for *Veratrum album* L. globules potencies of D2, D3, D4, and D6.

Ten differently potentiated globules samples of *Veratrum album* L. (D2, D3, D4, D6, D12, D30, D200) and *Veratrum viride var. viride* (D6, D12, D30) were analyzed by triplicate applying the validated MRM method. No alkaloids were detected in the *V. viride var. viride* globules as well as in the *V. album* L. globules potencies D6, D12, D30, D200. For the *V. album* L. globules, potencies of D2, D3, D4, and D6, chromatograms of the jervine quantifier and qualifier transitions are visualized in Figure 4. In the D2 sample, jervine produced a distinct peak without signal‐to‐noise interference. With increasing potency, signal‐to‐noise ratios became progressively more pronounced in the chromatograms, and peak intensities decreased accordingly. Between the D2 and D3 samples, the signal intensity decreases declined by approximately a factor of 10.

All measurements were performed in triplicate, and the averaged results for D2, D3, and D4 are presented in Table 5. Only jervine in the D2 globules exceeded the LOQ, with a concentration of 25 ng/g, allowing reliable quantification. The D2 samples were diluted prior to measurement and the concentration calculated using the dilution factor, ensuring full compliance with the validated linear calibration range of the method. In the D3 sample, jervine was detected between the LOQ and the LOD, confirming a positive verification and enabling only approximate quantification. The D4 sample showed jervine levels below the LOD, indicating trace amounts. However, a clear tenfold dilution effect is consistently evident across the D2–D4 samples.

**TABLE 5:** Analyzed jervine concentrations in *Veratrum album* L. globules.

*Veratrum album* L.	Jervine concentration
Globules potency	(ng/g)
D2	25.2
D3	Approximately 2.3
D4	Approximately 0.22 (traces)
D6	—

**FIGURE 5:**
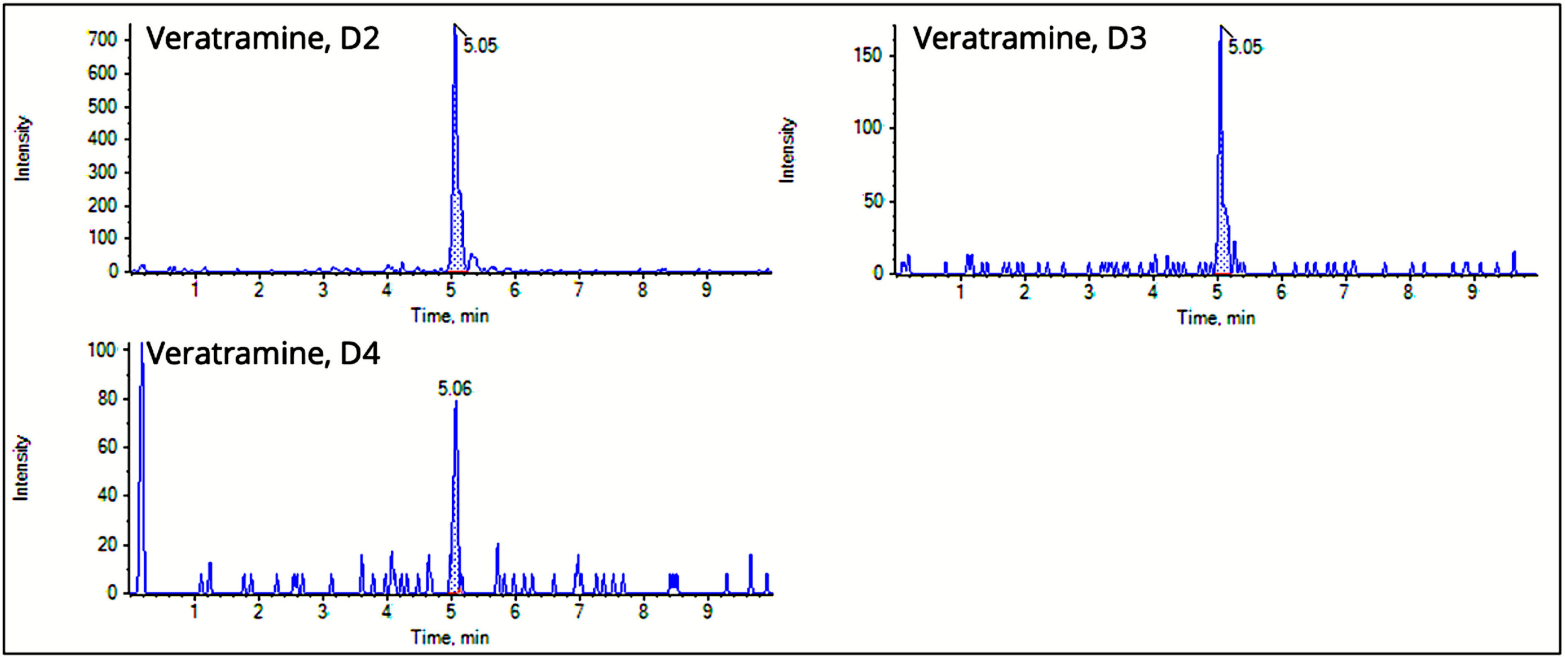
Chromatograms of veratramine quantifier (m/z 410 → 295) for *Veratrum album* L. globules potencies of D2, D3, and D4.

Protoveratrine A, cevadine, veratramine, and veratridine were not quantified in the globules. However, some clear peaks of veratramine in *V. album* L. globules of D2, D3, and D4 potencies were perceptible with decreasing intensities due to potentiation as depicted in Figure 5. The most plausible explanation is the differing natural abundances of the alkaloids in the *Veratrum* plant material (Siegle and Pietsch 2024). Additional contributing factors such as differing ionization efficiencies or stability during the globule preparation cannot be fully excluded.

According to the HAB, a mother tincture is extracted from the dried rhizome of *V. album* L., containing a declared minimum alkaloid content of 0.7% (calculated as protoveratrine A), as specified in regulation no. 33c (Bundesinstitut für Arzneimittel und Medizinprodukte 2023). Subsequent dilution steps with ethanol, following HAB regulations no. 4a or no. 19f, result in a tincture with a total alkaloid content ranging between 0.07% and 0.33% (Bundesinstitut für Arzneimittel und Medizinprodukte 2023). During the preparation of homeopathic globules, the respective dilutions are sprayed evenly onto the globule matrix, as described previously (Bundesinstitut für Arzneimittel und Medizinprodukte 2023). Assuming ideal mixing, homogeneous distribution, and adherence to the standard ratio of 1 part solution to 100 parts globules by mass, the theoretical alkaloid concentrations decrease by a factor of 10 with each decimal potency step. This serial tenfold decrease between potencies was consistent across our analytical results, supporting the assumption of uniform application and successful stepwise dilution. Furthermore, the absence of alkaloids in higher potencies (D6, D12, D30, D200) aligns with the principles of homeopathic preparation. The observed pattern confirms the integrity of the manufacturing process and demonstrates the capacity of the developed method to detect and track phytochemical residues even at the lower limits of homeopathic dilutions.

During studies of the teratogenicity of jervine, the lowest documented oral lethal dose LD_50_ in mice of 120 mg/kg was documented (Omnell et al. 1990). However, this toxicological value is outdated and difficult to apply for human purposes. Therefore, the consumption of an entire D2 globules bottle (10 g) has with high probability barely or mildly toxic effects (approximately 250 ng of jervine), but it cannot be conclusively clarified. This uncertainty is particularly concerning for small children, who might be exposed to these substances in products with sucrose formulations and inadequate childproof packaging.

A recent study examined 2512 exposure inquiries to the Erfurt Poison Information Center (2013–2022) related to botanical drug poisonings and found that most cases involved young children (Wendt et al. 2025). The Swiss Toxicological Information Center evaluates in a study the accidental ingestion of complementary and alternative medicine products by children between 1998 and 2007 (Zuzak et al. 2010). It reveals that poisonings due to homeopathic products (tablets, tinctures, globules, etc.) can indeed occur, albeit rarely and with mild symptoms (Zuzak et al. 2010). The simple use of homeopathic products is widely practiced in children for various health conditions, such as the potential treatment of unpleasant teething symptoms or sleep bruxism (Food and Drug Administration U.S. 2017a; Lombardi et al. 2019; Taneja et al. 2019; Tavares‐Silva et al. 2019). However, incidents such as the one in September 2016 involving homeopathic teething tablets and gels containing *Atropa belladonna* L. alkaloids demonstrate the importance of accurate analysis of such products (Food and Drug Administration U.S. 2017b)

The HAB utilized protoveratrine A, which was absent in this study, as a single reference alkaloid for the calculation of the overall alkaloid content (Bundesinstitut für Arzneimittel und Medizinprodukte 2023). Moreover, Buchholzer et al. (2019) also referred in the toxicological assessment of homeopathic products to protoveratrine A (acceptable amount per day = 0.06 μg/kg/d; Buchholzer et al. 2019) as toxicologically relevant matter and thereby, other alkaloids, such as jervine, were neglected.

Comparable literature is based mainly on the analysis of self‐made tinctures and less on commercially available homeopathic remedies. For example, a high‐performance thin layer chromatography (HPTLC) method was used to analyze and fingerprint 16 homeopathic mother tinctures that lack an official standard provided by the Homeopathic Pharmacopoeia of India (Kumar et al. 2025). Moreover, homeopathic mother tinctures of eight *Euphorbia* species were prepared using the methods outlined in the French Pharmacopoeia, and subsequently, their ingenol concentrations were analytically assessed via HPLC and ultraviolet (UV) detector (Girin et al. 1993). Additionally, plant compounds such as 6‐hydroxykynurenic acid (from *Ginkgo biloba* L., HPLC coupled with a diode array detector (DAD)) (Gräsel and Reuter 1998), strychnine (from *Strychnos nux‐vomica* L., HPLC‐UV) (Patel and Mashru 2017), cucurbitacin E and I (from *Gratiola officinalis* L., HPLC‐DAD) (Kaya and Melzig 2008) have been investigated in homeopathic preparations using HPLC methods. In previous studies, the alkaloid from *Berberis vulgaris* L., berberine, has been analyzed in homeopathic formulations (including gel; Soudagar et al. 2023) using HPLC coupled to DAD or UV detector (Chaudhari and Mashru 2017; Soudagar et al. 2023).

In summary, jervine was quantifiable in *V. album* L. D2 globules, detectable in D3, and present as trace amounts in D4. From potency D6 ( *V. album* L. and *V. viride var. viride*) onwards, no contents of the studied alkaloids were detected. Several challenges were faced, primarily due to the lack of analysis data of homeopathic products and reliable information on the toxicology of the individual substances examined. This lack of data hampers the ability to draw definitive conclusions about the safety and potential health risks of the individual substances and homeopathic products. Additionally, the content determination methods described in the HAB are incomparable to the robust and sensitive HPLC‐MS/MS method.

The toxicological relevance of these findings remains unclear due to the insufficient literature available on the subject. This uncertainty is especially troubling for small children, who may encounter these substances in sucrose‐based products that lack adequate childproof packaging. The lack of analytical approaches and conclusive toxicological data highlights the need for further research to ensure safety and prevent potential consumer health risks caused by homeopathic products.

## Funding

The authors have nothing to report.

## Conflicts of Interest

The authors declare no conflicts of interest.

## Data Availability

The data that support the findings of this study are available from the corresponding author upon reasonable request.
